# Lactate clearance is a predictor of sustained bleeding in emergency room patients with moderate upper gastrointestinal bleeding

**DOI:** 10.1186/cc12177

**Published:** 2013-03-19

**Authors:** T Wada, A Hagiwara, N Yahagi, A Kimura

**Affiliations:** 1 University of Tokyo, Japan; 2 National Center for Global Health and Medicine, Tokyo, Japan

## Introduction

There is no useful predictor of sustained upper gastrointestinal bleeding (UGIB). Glasgow-Blatchford scoring (GBS) is based on simple clinical and laboratory variables and can predict whether a UGIB patient needs an intervention or not. However, the intervention which the GBS mentions includes not only endoscopy but also blood transfusion. Therefore, we cannot determine whether a UGIB patient needs urgent endoscopy or just blood transfusion by GBS alone. We hypothesized that high lactate clearance (CLac) would decrease the likelihood of sustained UGIB.

## Methods

This is a retrospective study. UGIB patients, who visited the emergency department (ED) of the National Center for Global Health and Medicine from April 2011 to March 2012 and received urgent endoscopy in the ED, were enrolled. We collected for each patient the GBS, the blood lactate value on arrival in the ED, the blood lactate value after bolus administration of 20 to 40 ml/kg Ringer's acetate (initial fluid therapy) and the report of urgent endoscopy. We classified the severity of UGIB according to GBS. A score ≤12 was classified as moderate, and a score ≥13 was classified as severe. CLac was defined as the percentage decrease in blood lactate from the time of arrival in the ED to the time when an initial fluid therapy was finished. CLac <50% was defined as low, and CLac ≥50% was defined as high. Whether a patient had sustained bleeding or not was determined based on the report of urgent endoscopy. The relationship between CLac and sustained bleeding was examined by Fisher's exact test, and *P <*0.05 was considered statistically significant.

## Results

Seventy-nine patients were enrolled. Fifty-one patients were with moderate UGIB, and 28 patients were with severe UGIB. As indicated in Tables 1 and 2, there was a significant relationship between CLac and sustained bleeding in moderate UGIB (*P *= 0.02). On the other hand, there was no significant relationship between CLac and sustained bleeding in severe UGIB (*P *= 0.58).

**Table 1:** Relationship between lactate clearance and sustained bleeding in moderate UGIB (*n *= 51)

	Sustained bleeding (*n *= 19)	Nonsustained bleeding (*n *= 32)	*P *value
CLac <0.5	18 (95%)	21 (65%)	0.02
CLac ≥0.5	1 (5%)	11 (34%)	

**Table 2:** Relationship between lactate clearance and sustained bleeding in severe UGIB (*n *= 28)

	Sustained bleeding (*n *= 19)	Nonsustained bleeding (*n *= 32)	*P *value
CLac <0.5	4 (67%)	18 (82%)	0.58
CLac ≥0.5	2 (33%)	4 (18%)	

## Conclusion

If an initial fluid therapy for moderate UGIB results in high CLac, the bleeding might already have stopped. For such a patient, we may save urgent endoscopy.

